# Determination of Nutrients, Biomass, and Bacterial Quantification in Different Mangroves Sites: A Comparative Study on Nutrients Dependent Biomass Production

**DOI:** 10.1002/ece3.71697

**Published:** 2025-07-04

**Authors:** Sadar Aslam, You‐Shao Wang

**Affiliations:** ^1^ Laboratory of Tropical Oceanography, South China Sea Institute of Oceanology Chinese Academy of Sciences Guangzhou China; ^2^ Innovation Academy of South China Sea Ecology and Environmental Engineering Chinese Academy of Sciences Guangzhou China

**Keywords:** bacterial abundance, Guangdong China, Kaozhou Yang, mangrove ecosystem, nutrients availability, nutrients recycling via bacteria, total organic matter

## Abstract

This study was conducted at eight different sites of the mangrove ecosystem in Kaozhou Yang, Huidong District, Huizhou Guangdong, South China Sea. The concentration of nutrients (${\mathrm{NH}}_{4}^{+}$, ${\mathrm{NO}}_{2}^{-}$, ${\mathrm{NO}}_{3}^{-}$, ${\mathrm{PO}}_{4}^{3-}$ and ${\mathrm{SiO}}_{3}^{2-}$) was determined (both water and soil samples). Based on the nutrient concentration, the comparison of mangrove biomass production, total organic matter and bacterial count were also investigated. The level of nutrient values and biomass production of mangrove and bacterial count in both water and soil samples followed the same trend. The results showed that the highest concentration of ${\mathrm{NH}}_{4}^{+}$ (0.457 ± 0.051 mg/L), ${\mathrm{NO}}_{2}^{-}$ (0.223 ± 0.018 mg/L), ${\mathrm{NO}}_{3}^{-}$ (0.521 ± 0.038 mg/L), ${\mathrm{PO}}_{4}^{3-}$ = P (0.242 ± 0.049 mg/L) and ${\mathrm{SiO}}_{3}^{2-}$ = Si (4.094 ± 0. 095 mg/L) were found in the water samples from station S‐1, while the lowest values of ${\mathrm{NH}}_{4}^{+}$ (0.063 ± 0.007 mg/L), ${\mathrm{NO}}_{2}^{-}$ (0.0124 ± 0.001 mg/L), ${\mathrm{NO}}_{3}^{-}$ (0.053 ± 0.003 mg/L), ${\mathrm{PO}}_{4}^{3-}$ = P (0.012 ± 0.002 mg/L) and ${\mathrm{SiO}}_{3}^{2-}$ (0.713 ± 0.009 mg/L) were recorded in station S‐8. The order of nutrient values and bacterial count in the water and soil samples was same: S‐1 > S‐5 > S‐3 > S‐6 > S‐4 > S‐2 > S‐7 > S‐8. *Avicennia marina* was the only species found in all stations therefore; this species was considered for the assessment of the biomass of above‐ and belowground parts. The highest biomass (aboveground parts; 131.23 ± 2.09 Mg/ha, belowground parts; 139.86 ± 2.57 Mg/ha) was recorded at station S‐1. The lowest biomass (aboveground parts; 119.72 ± 1.99 Mg/ha, belowground parts; 127.13 ± 2.01 Mg/ha) was found at station S‐8. The analysis of organic matter (both water and soil samples) also showed the same trend. It was concluded that that mangrove biomass was nutrient‐dependent, confirming our hypothesis that “mangrove biomass could depend on the availability of nutrients.” Mangrove ecosystem plays an important role in coastal and marine food webs and is closely connected to the well‐being of coastal communities. Therefore, the mangrove ecosystem is mainly included in the United Nations Sustainable Development Goals (SDGs) and the Paris Agreement (climate change mitigation) in this decade. This study is in line with SDGs 12 (Responsible consumption and production of food), 13 (Climate action), 14 (Life below water) and 17 (Partnerships with the goals). The mangrove plants convert carbon dioxide (toxic form of carbon) in its useful form (biomass), and in addition, the mangrove ecosystem serves as a food and nursery area for fish and shellfish fisheries. Therefore, this research promotes the role of the mangrove ecosystem to benefit the blue economy and mitigate climate change. It was concluded that the abundance of bacteria and the biomass of mangroves depend on the availability of nutrients. Therefore, the results of this study strengthen our hypothesis. In the future, this study could serve as a reference study for blue carbon sequestration in the mangrove ecosystem.

## Graphical abstract

The present study is hypothesis based that “mangrove biomass depends on the availability of nutrients in the mangrove ecosystem” and a baseline data from the selected locations. This research aligns with the Paris agreement and Sustainable Development Goals (SDG) 12, 13, 14, and 17. This research is favorable for promoting the role of mangrove ecosystem in the benefit of blue economy. Mangroves areas with high nutrients and bacterial abundance, in near intact ecosystems are highlighted as a key policy to mitigate for both the climate and biodiversity. Through this research the government could potentially draw attention to these future strategy for sustainable management of the mangrove forests.

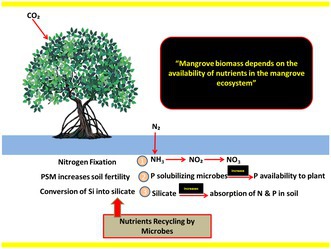

## 1 Introduction

Mangrove forests are the most unique ecosystem of halophytic shrubs, trees, and other woody floras that grow in the intertidal zone of subtropical and tropical coastal areas. Mangrove plants have four exclusive characteristics: return rate (the CO_2_ removed from the environment by photosynthesis), productivity, decomposition rate, resilience to extreme climate, and anthropogenic activities. Therefore, mangroves represented globally as the most unique marine ecosystem (Wang 2019; Wang and Gu 2021). Due to their high biomass production and slow decomposition rate, mangrove forests are considered one of the most carbon‐rich ecosystems (Donato et al. 2011; Alongi 2014). Carbon storage capacity in mangrove forests is persistently higher as compare to other tropical forests (Kauffman and Donato 2012; Bai et al. 2021). Due to their high carbon storage capacity, environmentalists have increased their interest in mangrove ecosystems in recent decades (Atwood et al. 2017; Doughty et al. 2016; Hutchison et al. 2014; Rovai et al. 2018; Bai et al. 2021; Niu et al. 2024). Mangrove ecosystems are also considered the most important hotspots for the sequestration of inorganic carbon (Donato et al. 2011). They are able to bind the nutrients in the soil/sediment and regulate nutrient fluxes between water and sediments (Constance et al. 2022). Mangroves also play a significant role in neutralizing the toxic (inorganic) form of carbon dioxide (in the environment) into potential biomass (Waramit et al. 2023).

The mangrove ecosystem supports a diverse food web (Sievers et al. 2019; Zhao et al. 2020) and provides high nutrient availability controlled by various (biotic and abiotic) factors, e.g., tidal inundation, soil type, elevation in the tidal frame, litter production, decomposition, and microbial activity (Reef et al. 2010). Microbes consume the decaying material (leaf litter, etc.) and convert it into recycled nutrients such as nitrogen, phosphorus, etc., which increase soil fertility and play a key role in mangrove growth (Pradisty et al. 2021). Therefore, the microorganisms in the soil play an important role in improving nutrient availability, an acquirement for the plants growth (Alori et al. 2017). These microorganisms convert the dissolved and unusable form of the elements into useful nutrients through a variety of biological processes. One of the most important biological processes is nitrogen fixation, in which nitrogen (N_2_) is converted into ${\mathrm{NH}}_{4}^{+}$, ${\mathrm{NO}}_{2}^{-}$, and ${\mathrm{NO}}_{3}^{-}$ (Bernhard 2010). Some microbes solubilize and mineralize the insoluble soil phosphorus, while other types of microorganisms in the rhizosphere and soil are able to discharge phosphorus from the total phosphorus content of the soil through various processes, such as mineralization and solubilization (Bhattacharyya and Jha 2012). These types of microorganisms are called phosphorus solubilizing microorganisms (PSM). They mobilize phosphorus and make it available to plants through solubilization and mineralization processes (Zhu et al. 2011; Alori et al. 2017). Therefore, the mangrove ecosystem plays an important role in maintaining the biogeochemical cycling of phosphorus and nitrogen (via the bacterial community); it also maintains the distribution of biogenic components (Yang et al. 2016; Feng et al. 2017, 2019).

Mangrove ecosystem is considered hotspots for the Si cycle as they play a crucial role in plant growth and the transport, processing, and recycling of other nutrients (Elizondo et al. 2021). It also supports other biogeochemical cycles by efficiently absorbing and storing Si in plant parts and then also releasing it back into the soil/sediments (Hou et al. 2010; Liang et al. 2015). Si is also involved in the decomposition of organic matter, carbon storage, and nutrient cycling (Xia et al. 2020). This biogeochemical process of Si in the mangrove ecosystem also determines the exchangeable amount and form of other nutrients available to the coastal ecosystem (Wang et al. 2023). For example, the increase in Si availability leads to better accessibility of P and N in the soil. Thus, Si has a positive effect on plant growth and soil fertility (Pavlovic et al. 2021; Uhuegbue et al. 2024).

The present study was based on the hypothesis; “mangrove biomass could depend on the availability of the nutrients”. Therefore, the first part of the study assessed nutrients at the different mangrove sites, and the next part of our study involved the quantification of bacteria and the assessment of mangrove biomass production. In this study, ammonium (${\mathrm{NH}}_{4}^{+}$), nitrite (${\mathrm{NO}}_{2}^{-}$), nitrate (${\mathrm{NO}}_{3}^{-}$), phosphate (${\mathrm{PO}}_{4}^{3-}$ = P) and silicate (${\mathrm{SiO}}_{3}^{2-}$ = Si) were determined in sediments and water samples. Biomass production (of the mangrove) and total organic matter in both water and soil samples were determined in comparison to nutrient concentrations. The bacterial count/abundance in the mangrove ecosystem was also determined using flow cytometry.

## 2 Materials and Methods

### 2.1 Study Area

The study area was the Kaozhou Yang (22°43′56.20″N and 114°53′56.40″E), Huidong County, located in the southeast of Guangdong Province, China, with a section of the South‐China Sea coast. It is under the administration of the Huizhou Mangrove Municipal Nature Reserve. This area is a relatively developed, urbanized place and is under pressure from human activities. The maximum temperature in this area is 30°C and the minimum temperature is 17°C. The rainy season of the year lasts 7.3 months, from April 5 to November 16, with a sliding 31‐day rainfall of at least 0.5 inches. The rainiest month in Huidong is July, with an average rainfall of 7.3 inches. The month with the least rainfall in Huidong is December, with an average rainfall of 0.1 inches. Temperature, pH, salinity, conductivity, TDS, and DO are listed in Table 1 while the soil texture of the study sites is listed in Table 2. There are eight mangrove species present at these sites: *Avicennia marina* (common in all sites), *Rhizophora stylosa, Bruguiera gymnorhiza, Sonneratia apetala, Laguncularia racemosa, Acanthus ilicifolius, Kandelia obovata*, and *Excoecaria agallocha* . Most of the mangroves on the Huidong coast are man‐made planted, so the mangrove cover is not very high. Some mangroves are surrounded by the cultivation zones. The quality of community culture and awareness about protection of the environment both are underdeveloped. Due to a growing population, industrial development, and over‐construction, the beauty of the entire nature of the mangrove reserves is obscured (Wang et al. 2021). Eight different stations (S‐1 to S‐8) were selected from the mangrove area of Kaozhou Yang, Huidong Bay along the South China Sea. Sites 1 to 6 were close to urban populations, so these sites were highly affected by human activity, while sites 7 and 8 were rural areas with little human activity. Different zones (low, medium, and high) were selected for sampling. The low zone was within the mangrove ecosystem, the medium zone was 200 m away from the mangroves (toward the open sea), while the high zone was 400 m away from the mangrove ecosystem (toward the open sea). Distance (from the mangrove area) was chosen to assess nutrient distribution as a function of distance from the mangrove area (and comparison of nutrients within the mangrove area). The size or number of samples was determined according to the analysis, and to obtain a better statistical approach, each sample analysis was repeated (*n* = 9).

**TABLE 1:** Water quality parameters at study areas.

Parameters	S1	S2	S3	S4	S5	S6	S7	S8
Temperature (°C)	25.02 ± 0.01^*c*^	29.13 ± 0.32^*a*^	28.33 ± 0.06^*a*^	28.77 ± 0.06^*a*^	28.50 ± 0.35^*a*^	27.80 ± 0.10^*b*^	29.90 ± 0.10^*a*^	29.13 ± 0.15^*a*^
pH	7.05 ± 0.01^*a*^	7.04 ± 0.01^*a*^	7.05 ± 0.01^*a*^	7.05 ± 0.01^*a*^	6.95 ± 0.04^*b*^	7.04 ± 0.01^*a*^	7.04 ± 0.03^*a*^	7.03 ± 0.03^*a*^
Salinity (ppt)	31.69 ± 0.02^*a*^	34.43 ± 0.15^*c*^	33.09 ± 0.02^*b*^	33.96 ± 0.01^*b*^	32.35 ± 0.08^*a*^	33.64 ± 0.10^*b*^	35.77 ± 0.01^*d*^	35.38 ± 0.07^*d*^
TDS (mg/L)	6.50 ± 0.02^*b*^	3.04 ± 0.12^*c*^	11.62 ± 0.99^*a*^	2.61 ± 0.06^*b,c*^	5.43 ± 0.06^*b*^	5.58 ± 0.06^*b*^	3.32 ± 0.01^*b*^	0.23 ± 0.06^*d*^
DO (mg/L)	7.06 ± 0.01^*a*^	6.58 ± 0.01^*a,b*^	7.61 ± 0.06^*a*^	7.64 ± 0.05^*a*^	7.12 ± 0.06^*a*^	7.13 ± 0.06^*a*^	7.47 ± 0.12^*a*^	8.22 ± 0.02^*a*^

*Note:* Data are represented as mean ± SD (*N* = 9). ± SD showing standard deviation while letters (superscripts) indicate significant differences among variables (*a* = *p* < 0.05; *b* = *p* < 0.01; *c, d* = *p* < 0.001).

**TABLE 2:** Grain size composition (sand and silt/clay fraction) at study areas.

Sites	S1	S2	S3	S4	S5	S6	S7	S8
Sand	0.60 ± 0.06^*d*^	3.26 ± 0.01^*a*^	2.10 ± 0.03^*b*^	3.17 + 0.02^*a*^	1.01 ± 0.03^*c*^	3.15 ± 0.02^*a*^	3.61 ± 0.24^*a*^	3.80 ± 0.19^*a*^
Silt	9.22 + 0.01^*a*^	6.16 ± 0.02^*b*^	7.89 ± 0.02^*b*^	6.51 ± 0.07^*b*^	9.02 ± 0.07^*a*^	7.21 ± 0.33^*b*^	5.92 ± 0.21^*c*^	5.91 + 0.22^*c*^
Clay	1.11 ± 0.03^*a*^	0.62 ± 0.03^*b*^	0.83 ± 0.09^*a*^	0.63 ± 0.15^*b*^	0.93 ± 0.02^*a*^	0.67 ± 0.07^*b*^	0.56 ± 0.15^*b*^	0.20 ± 0.05^*c*^

*Note:* Data are represented as mean ± SD (*N* = 9). ± SD showing standard deviation while letters (superscripts) indicate significant differences among variables (*a* = *p* < 0.05; *b* = *p* < 0.01; *c* = *p* < 0.001).

**FIGURE 1:**
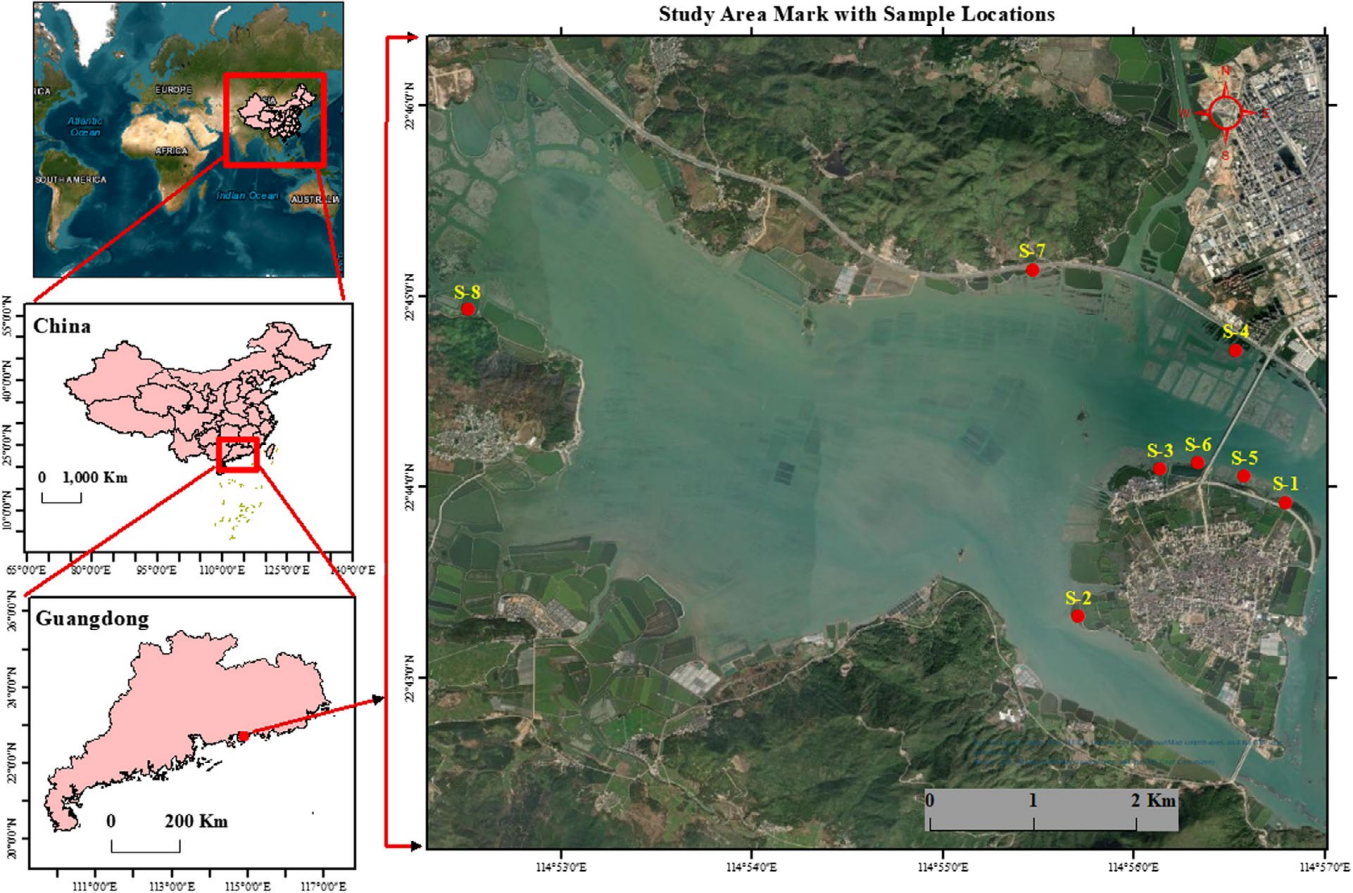
Map of eight study stations (S‐1 to S‐8) in Kaozhou Yang, Huidong County, Guangdong Province, China.

The study area map is shown in Figure 1. A survey was conducted in these areas in April to May 2024.

### 2.2 Determination of Nutrients by Spectrophotometric Analysis

The determination of ammonium, nitrate, nitrite, phosphate, and silicate was done by using spectrophotometric analysis (see the Appendix S2). For the preparation of reagents and standard solutions, the double distilled deionized water was used.

### 2.3 Determination of Bacterial Count by Flow Cytometric (FCM) Technique

The bacterial cells in the samples (see Appendix S2 for sample collection and preparation) were stained with SYBR Green I in anhydrous dimethyl sulfoxide (DMSO). After addition of these reagents, suspension (sample) was incubated in the dark for 15 min. After incubation, the samples were diluted with saline sample (1%) and filtered using a Millex‐GP filter with a pore size of 0.22 μm. The samples were then ready for analysis using a CyFlow space flow cytometer system (Beckman Coulter, United States) equipped with a 200‐mW solid‐state laser emitting light at 488 nm. Green and red fluorescence were measured at 520 nm (FL1 channel) and 630 nm (FL3 channel). The flow cytometer was set as in the following follows: gain FL1 = 495, gain FL3 = 50, speed = 4 (implying an event rate never exceeding 1000 events per second). Counts were recorded as logarithmic signals and were triggered on the green fluorescence channel (FL1). Data were processed with CytExpert 2.1, using electronic gating to separate the desired events. Presentation of the data as FL1/FL3 dot plots allowed for optimal distinction between stained intact microbial cells and instrument noise or sample background (Frossard et al. 2016).

### 2.4 Biomass Estimation

The total biomass of above‐ and belowground parts of *Avicennia marina* was estimated. *A. marina* was found at all sampling sites (S‐1 to S‐8); therefore, this species was selected for the biomass analysis. A 10 m × 10 m transect was selected to calculate the biomass of *A. marina* . The details of the calculation of the above‐ground and below‐ground plant biomass are explained in the following sections.

### 2.5 Aboveground Biomass

The aboveground biomass was estimated by adding the total biomass of above ground parts (stem, branch, and leaf). The stem volume was calculated using Newton's formula. The wood was obtained by boring the corer into the stem at a depth of 7.5 cm, and the specific gravity (G) of the stem was determined. The moisture content of this piece of wood was removed by drying it in an oven at 70°C for 12 h, and the specific gravity was calculated (dividing mass and volume). Subsequently, the biomass (BS) of the stem was calculated using the following formula (Kauffman and Donato 2012; Mitra and Zaman 2014; Patil et al. 2014):

BS=G.V

To determine the biomass of the branches, the total number of branches of the plants was counted. Based on their diameter (< 6 cm, 6–10 cm, and > 10 cm), the branches were divided into three groups. The branches were cut, and the leaves on the branches were removed. To remove the moisture content, the branches were dried in an oven at 70°C for 12 h. The biomass of two branches from each size group was determined separately using the following equation (Mitra and Zaman 2014; Sitoe et al. 2014):

$$B_{\mathrm{db}}=n_{1}{\mathrm{bw}}_{1}+n_{2}{\mathrm{bw}}_{2}+n_{3}{\mathrm{bw}}_{3}=\sum n_{i}{\mathrm{bw}}_{i}$$

where *B*_*db*_ is the biomass of the plant, *n*_*i*_ is the number of branches in the branch of each group, *bw*_*i*_ is the average weight of branches of each group, and *i* = 1, 2, 3, …, *n* are the branch groups. The biomass was finally calculated by multiplying by the number of plants.

All leaves from the branches (three different groups) of each tree were removed and dried in the oven at 70°C, and the dry weight was calculated. The biomass of the leaves of each tree was then calculated by multiplying the average biomass of leaves per branch by the number of branches in that tree using the following equation (Mitra and Zaman 2014):

$$L_{\mathrm{db}}=n_{1}\times {\mathrm{Lw}}_{1}+n_{2}\times {\mathrm{Lw}}_{2}+\ldots +n_{i}\times {\mathrm{Lw}}_{i}$$

Where *L*_*db*_ is the biomass of leaves, *n* is the number of branches of each tree in the *i*th branch group, and *Lw* is the average dry weight of leaves removed from the branches.

### 2.6 Belowground Biomass Estimation

The allometric equation proposed by Dharmawan and Siregar (2008) was used to calculate the biomass of belowground parts (roots/pneumatophores).

$$W_{r}=\frac{0}{1682}\times {\left(\mathrm{DBH}\right)}^{1/7939}$$

where *W*_*r*_ is the biomass of below‐ground parts (in Kg), DBH is the diameter at breast height (in cm).

### 2.7 Pneumatophores Biomass Estimation

The pneumatophores of *A. marina* were counted in the specific area/microplot (50 × 50 cm). After counting, the pneumatophores were removed and dried in the oven. The mass (mean value) of the oven‐dried samples of pneumatophores was calculated by multiplying by the number of pneumatophores in the specific area (square 50 × 50 cm) and the calculation was performed per hectare of area (Kauffman and Donato 2012; Sitoe et al. 2014).

### 2.8 Estimate of Soil and Water Organic Matter by Weight Loss on Ignition (LOI) Method

The loss on ignition (LOI) method was used to determine the organic matter in the sediments. This method was based on sequential heating of the samples in a muffle furnace. In this method, the sediment samples were dried in the oven for 12–24 h (until a constant weight was reached) at 105°C. The dried samples were then burned for 8 h at 550°C in the muffle furnace. For the water samples, 100 mL of the water sample was filtered through Whatman GF/C filters (1.2 μm). To remove NaCl, the water samples were rinsed with distilled water. The samples were then dried for 12 h at 60°C and weighed. The dried filter paper, which contained all the organic matter, was then burnt to ash at 450°C for 3 h and the mass of the total organic matter was then determined (Heiri et al. 2001).

### 2.9 Data Analysis

All the data found from the present analysis was presented in mean ± SD (*n* = 9). The findings were investigated by using Microsoft Excel (ver. 16.67) and MANOVA (Multivariate Analysis of Variance) was performed through SPSS (ver. 28.0_ Chicago, IL, USA) followed by Post Hoc Tukey test. The statistical analysis was site specific (carried out on the variables, and the comparison was conducted among the sites). For the analysis of the nutrients in the water samples, the sampling points were divided into low, med, and high zones, so a comparison of the individual values between the zones (low, med, and high) was carried out. The values had been verified for the homogeneity in variation prior to significant investigation. All values of *p* < 0.05 were considered statistically significant. A hand‐held GPS (Global Positioning System) device with standard accuracy ±0.3 m (“Garmin GPS Map 66 s Rugged Multisatellite with Sensors 3” Color Display) was used to collect and ID site locations by using ArcGIS (ver. 10.7.1) software for mapping in Figure 1.

## 3 Results

**FIGURE 2:**
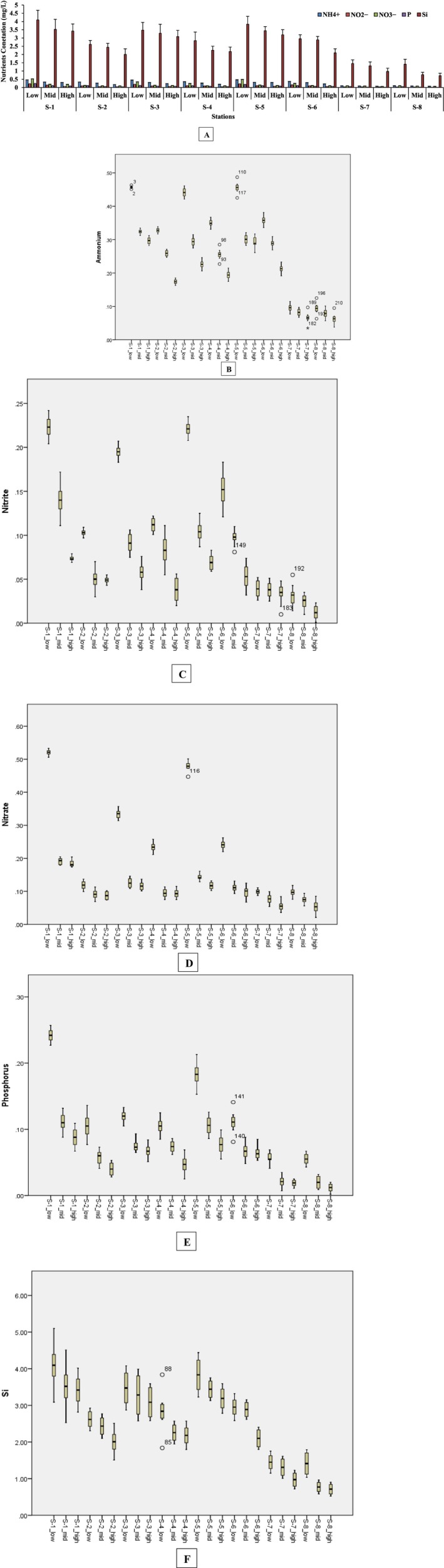
Bar graph (A) and boxplot comparisons of ${\mathrm{NH}}_{4}^{+}$ (B), ${\mathrm{NO}}_{2}^{-}$ (C), ${\mathrm{NO}}_{3}^{-}$ (D), P (E), and Si (F) for nutrients concentration in water (low, mid, high) samples of eight stations (S‐1 to S‐8).

**FIGURE 3:**
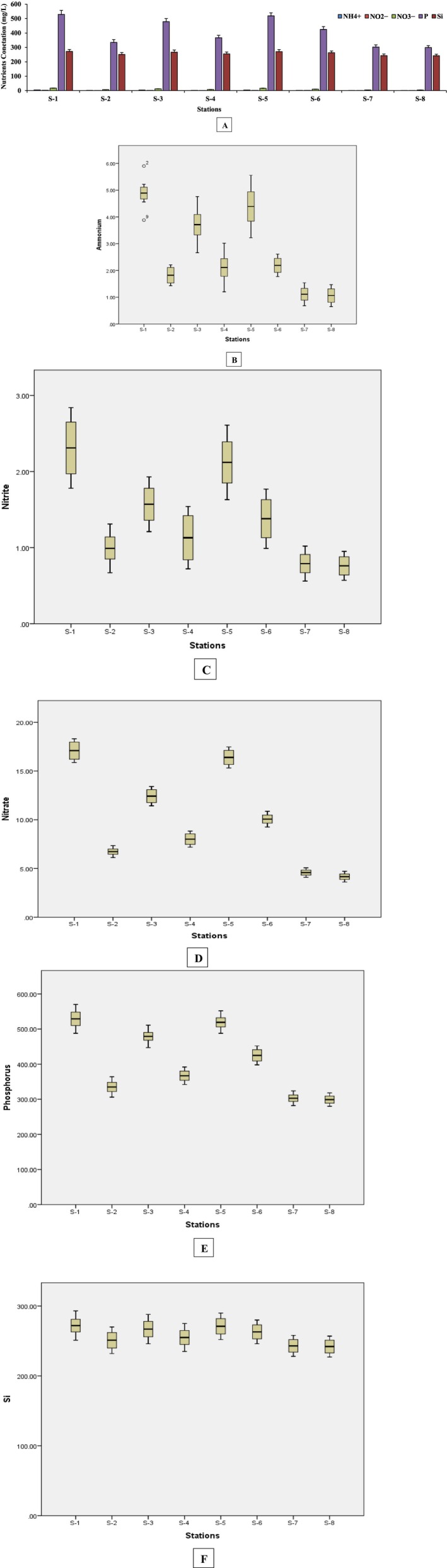
Bar graph (A) and boxplot comparisons of ${\mathrm{NH}}_{4}^{+}$ (B), ${\mathrm{NO}}_{2}^{-}$ (C), ${\mathrm{NO}}_{3}^{-}$ (D), P (E), and Si (F) for nutrients concentration in soil samples of eight stations (S‐1 to S‐8).

In the present study, the comparison of biomass production in the mangrove ecosystem with the nutrients (${\mathrm{NH}}_{4}^{+}$, ${\mathrm{NO}}_{2}^{-}$, ${\mathrm{NO}}_{3}^{-}$, P, and Si) and the abundance of bacteria (by flow cytometry) found in the eight stations S‐1 to S‐8 (Figure 1), were investigated. To determine the concentration of nutrients in the water samples (Table 3A, 3B), it was found that the highest concentration of ${\mathrm{NH}}_{4}^{+}$ (0.457 ± 0.051 mg/L); ${\mathrm{NO}}_{2}^{-}$ (0.223 ± 0.018 mg/L); ${\mathrm{NO}}_{3}^{-}$ (0.521 ± 0.038 mg/L); P (0.242 ± 0.049 mg/L) and ${\mathrm{SiO}}_{4}^{2-}$ (4.094 ± 0. 095 mg/L) were found in the water samples from station S‐1, while the lowest values of ${\mathrm{NH}}_{4}^{+}$ (0.063 ± 0.007 mg/L), ${\mathrm{NO}}_{2}^{-}$ (0.0124 ± 0.001 mg/L), ${\mathrm{NO}}_{3}^{-}$ (0.053 ± 0.003 mg/L), P (0.012 ± 0.002 mg/L) and Si (0.713 ± 0.009 mg/L) were recorded in station S‐8 (Figure 2). The same pattern of nutrient values was observed in the soil samples (Table 4). The highest values of ${\mathrm{NH}}_{4}^{+}$ (4.89 ± 0.15 mg/Kg), ${\mathrm{NO}}_{2}^{-}$ (2.31 ± 0.17 mg/Kg), ${\mathrm{NO}}_{3}^{-}$ (17.09 ± 1.01 mg/Kg), P (529 ± 17.95 mg/Kg) and ${\mathrm{SiO}}_{3}^{2-}$ (272 ± 12.41 g/Kg) were recorded in S‐1, while the lowest (${\mathrm{NH}}_{4}^{+}$; 1. 06 ± 0.3 mg/Kg, ${\mathrm{NO}}_{2}^{-}$; 0.76 ± 0.05 mg/Kg, ${\mathrm{NO}}_{3}^{-}$; 4.16 ± 0.49 mg/Kg, P; 299 ± 15.16 mg/Kg, and ${\mathrm{SiO}}_{3}^{2-}$; 242 ± 13.76 g/Kg) were found in the station S‐8 samples of soil (Figure 3). The order of nutrient values in the water and soil samples was the same: S‐1 > S‐5 > S‐3 > S‐6 > S‐4 > S‐2 > S‐7 > S‐8. Calibration curve for these nutrients have been shown in Figure S1.

**TABLE 3A:** Nutrients (${\mathrm{NH}}_{4}^{+}$, ${\mathrm{NO}}_{2}^{-}$, ${\mathrm{NO}}_{3}^{-}$, P, Si) analysis of seawater samples from eight stations (S‐1 to S‐4)

	S‐1			S‐2			S‐3			S‐4		
Station with zone	Low	Mid	High	Low	Mid	High	Low	Mid	High	Low	Mid	High
${\mathrm{NH}}_{4}^{+}$ (mg/L)	0.457 ± 0.004^a^	0.324 ± 0.007^b^	0.297 ± 0.010^b^	0.328 ± 0.007^b^	0.259 ± 0.009^b^	0.174 ± 0.007^c^	0.441 ± 0.013^a^	0.290 ± 0.013^b^	0.226 ± 0.012^b,c^	0.349 ± 0.011^b^	0.256 ± 0.016^b^	0.194 ± 0.012^c^
${\mathrm{NO}}_{2}^{-}$ (mg/L)	0.223 ± 0.012^a^	0.140 ± 0.019^b^	0.074 ± 0.003^c^	0.103 ± 0.004^b^	0.050 ± 0.012^c^	0.049 ± 0.004^c^	0.195 ± 0.007^a^	0.091 ± 0.0012^c^	0.058 ± 0.011^c^	0.112 ± 0.008^b^	0.083 ± 0.019^c^	0.038 ± 0.013^c^
${\mathrm{NO}}_{3}^{-}$ (mg/L)	0.521 ± 0.009^a^	0.192 ± 0.010^c^	0.185 ± 0.010^c^	0.119 ± 0.013^c^	0.091 ± 0.014^d^	0.087 ± 0.012^d^	0.335 ± 0.014^b^	0.125 ± 0.014^c^	0.116 ± 0.013^c^	0.234 ± 0.014^c^	0.094 ± 0.013^d^	0.093 ± 0.013^d^
P (mg/L)	0.242 ± 0.010^a^	0.110 ± 0.013^b^	0.088 ± 0.014^b^	0.105 ± 0.019^b^	0.058 ± 0.012^c^	0.040 ± 0.010^c^	0.120 ± 0.009^b^	0.075 ± 0.009^b^	0.067 ± 0.011^b,c^	0.105 ± 0.012^a^	0.074 ± 0.009^b^	0.047 ± 0.013^c^
Si (mg/L)	4.094 ± 0.590^a^	3.520 ± 0.610^a^	3.419 ± 0.434^a^	2.616 ± 0.236^b^	2.435 ± 0.244^b^	2.007 ± 0.388^b^	3.476 ± 0.468^a^	3.283 ± 0.554^a^	3.086 ± 0.384^a^	2.838 ± 0.523^b^	2.256 ± 0.249^b^	2.181 ± 0.271^b^

*Note:* S‐1 to S‐4; different mangroves stations. Low zone was within the mangroves ecosystem and mid zone was 200 m away from the mangroves (toward the open sea) while high zone was 400 m away from the mangrove ecosystem (toward the open sea). $P={\mathrm{PO}}_{4}^{3-}$ and $\mathrm{Si}={\text{SiPO}}_{3}^{2-}$. ± SD showing standard deviation while data (means ± SD, *n* = 9) followed by different letters (superscripts) indicate significant differences among variables (a = *p* < 0.05; b = *p* < 0.01; c and d = *p* < 0.001)

**TABLE 3B:** Nutrients (${\mathrm{NH}}_{4}^{+}$, ${\mathrm{NO}}_{2}^{-}$, ${\mathrm{NO}}_{3}^{-}$, P, Si) analysis of seawater samples from eight stations (S‐5 to S‐8)

	S‐5			S‐6			S‐7			S‐8		
Station with zone	Low	Mid	High	Low	Mid	High	Low	Mid	High	Low	Mid	High
${\mathrm{NH}}_{4}^{+}$ (mg/L)	0.456 ± 0.017^a^	0.301 ± 0.013^b^	0.291 ± 0.018^b^	0.358 ± 0.013^b^	0.289 ± 0.012^b^	0.213 ± 0.013^c^	0.096 ± 0.011^d^	0.082 ± 0.010^d^	0.066 ± 0.016^d^	0.094 ± 0.017^d^	0.079 ± 0.014^d^	0.063 ± 0.016^d^
${\mathrm{NO}}_{2}^{-}$ (mg/L)	0.221 ± 0.008^a^	0.104 ± 0.012^b^	0.069 ± 0.009^c^	0.152 ± 0.021^b^	0.098 ± 0.009^c^	0.053 ± 0.013^c^	0.039 ± 0.010^d^	0.038 ± 0.009^d^	0.033 ± 0.012^d^	0.032 ± 0.012^d^	0.025 ± 0.008^d^	0.012 ± 0.008^d^
${\mathrm{NO}}_{3}^{-}$ (mg/L)	0.479 ± 0.015^a^	0.142 ± 0.010^c^	0.117 ± 0.011^c^	0.241 ± 0.014^c^	0.111 ± 0.012^c,d^	0.096 ± 0.019^d^	0.099 ± 0.007^d^	0.077 ± 0.014^d^	0.055 ± 0.014^d^	0.097 ± 0.013^d^	0.075 ± 0.012^d^	0.053 ± 0.019^d^
P (mg/L)	0.183 ± 0.017^a^	0.106 ± 0.014^b^	0.077 ± 0.014^b^	0.111 ± 0.017^b^	0.067 ± 0.014^b,c^	0.064 ± 0.011^b,c^	0.056 ± 0.008^c^	0.021 ± 0.009^d^	0.019 ± 0.005^d^	0.055 ± 0.009^c^	0.020 ± 0.009^d^	0.012 ± 0.006^d^
Si (mg/L)	3.834 ± 0.472^a^	3.441 ± 0.253^a^	3.190 ± 0.317^a^	2.953 ± 0.242^b^	2.887 ± 0.211^b^	2.102 ± 0.242^b^	1.451 ± 0.219^c^	1.311 ± 0.241^c^	0.973 ± 0.197^c^	1.414 ± 0.297^c^	0.773 ± 0.149^d^	0.713 ± 0.152^d^

*Note:* S‐5 to S‐8; different mangroves stations. Low zone was within the mangroves ecosystem and mid zone was 200 m away from the mangroves (toward the open sea) while high zone was 400 m away from the mangrove ecosystem (toward the open sea). $P={\mathrm{PO}}_{4}^{3-}$ and $\mathrm{Si}={\text{SiPO}}_{3}^{2-}$. ± SD showing standard deviation while data (means ± SD, *n* = 9) followed by different letters (superscripts) indicate significant differences among variables (a = *p* < 0.05; b = *p* < 0.01; c and d = *p* < 0.001).

**TABLE 4:** Nutrients (${\mathrm{NH}}_{4}^{+}$, ${\mathrm{NO}}_{2}^{-}$, ${\mathrm{NO}}_{3}^{-}$, P, Si) analysis of the soil samples from the mangrove ecosystem of eight stations (S‐1 to S‐8).

Station with zone	S‐1	S‐2	S‐3	S‐4	S‐5	S‐6	S‐7	S‐8
${\mathrm{NH}}_{4}^{+}$ (mg/L)	4.89 ± 0.55^*a*^	1.82 ± 0.32^*c*^	3.71 ± 0.63^*a*^	2.11 ± 0.62^*b*^	4.39 ± 0.79^*a*^	2.19 ± 0.31^*b*^	1.11 ± 0.30^*d*^	1.06 ± 0.30^*d*^
${\mathrm{NO}}_{2}^{-}$ (mg/L)	2.31 ± 0.41^*a*^	0.99 ± 0.21^*c*^	1.57 ± 0.26^*b*^	1.13 ± 0.31^*b*^	2.12 ± 0.34^*a*^	1.38 ± 0.28^*b*^	0.79 ± 0.16^*c*^	0.76 ± 0.14^*c*^
${\mathrm{NO}}_{3}^{-}$ (mg/L)	17.09 ± 1.94^*a*^	06.73 ± 0.39^*c*^	12.42 ± 0.71^*b*^	08.01 ± 0.64^*c*^	16.39 ± 0.79^*a*^	10.06 ± 0.58^*b*^	04.57 ± 0.35^*d*^	04.16 ± 0.38^*d*^
P (mg/L)	529 ± 28.23^*a*^	335 ± 19.36^*b*^	479 ± 20.95^*a*^	367 ± 17.46^*b*^	519 ± 20.71^*a*^	425 ± 19.30^*b*^	303 ± 14.55^*c*^	299 ± 13.56^*c*^
Si (mg/L)	272 ± 13.89^*a*^	251 ± 13.26^*a*^	267 ± 14.80^*a*^	255 ± 13.65^*a*^	271 ± 13.88^*a*^	263 ± 12.32^*a*^	243 ± 10.90^*a*^	242 ± 10.71^*a*^

*Note:* P = ${\mathrm{PO}}_{4}^{3-}$ and Si = ${\mathrm{SiO}}_{3}^{2-}$. ± SD showing standard deviation while data (means ± SD, *n* = 9) followed by different letters (superscripts) indicate significant differences among variables (*a* = *p* < 0.05; *b* = *p* < 0.01; *c, d* = *p* < 0.001).

**FIGURE 4:**
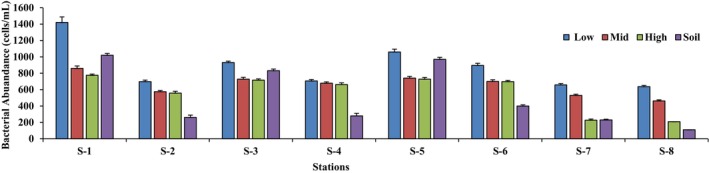
Bacterial abundance (cells/mL) in water (low, mid, high) and soil samples of eight stations (S‐1 to S‐8).

In this study, the total bacterial abundance at the eight stations (S‐1, S‐2, S‐3, S‐4, S‐5, S‐6, S‐7 and S‐8) was also determined by flow cytometry (Table 5). The results (seawater) showed that in each station, bacterial abundance was highest in the low zone, while the lowest bacterial abundance value was recorded in the high zone. When comparing the bacterial abundance of all seawater samples from all stations, the order of bacterial abundance was: S‐1 > S‐5 > S‐3 > S‐6 > S‐4 > S‐2 > S‐7 > S‐8 (Figure 4). A similar pattern of the bacterial abundance was determined for the soil samples. The results indicated that the pattern of bacterial count/abundance (in both water and soil samples) was the same in all stations as in the nutrient analysis. Bacterial abundance was found to be nutrient dependent. The highest nutrients concentration promoted the highest bacterial count, while the mangroves ecosystem with the lowest nutrients concentration had the lowest bacterial count. The images of bacterial abundance are given in the Figure S2 (1–8) for water samples and Figure S3 for soil samples. The highest value of bacterial abundance (1.42 × 10^8^ ± 88.43 cells/mL) was recorded in station S‐1, while the lowest value (2.09 × 10^7^ ± 3.75 cells/mL) was recorded in station S‐8. The bacterial abundance in soil samples was highest (1.02 × 10^10^ ± 1.12 cells/mL) in station S‐1, and the lowest value (1.1 × 10^9^ ± 1.90 cells/mL) was recorded in the soil samples from station S‐8.

**TABLE 5:** Bacterial abundance at eight stations (S‐1 to S‐8) in water and soil samples.

Zones	S‐1	S‐2	S‐3	S‐4	S‐5	S‐6	S‐7	S‐8
**Bacterial abundance (cells/mL) in water**								
Low	1.42 × 10^8^ ± 67.62^*a*^	6.98 × 10^7^ ± 18.13^*c*^	9.31 × 10^7^ ± 15.70^*b*^	7.06 × 10^7^ ± 17.18^*b*^	1.06 × 10^8^ ± 33.67^*a*^	8.96 × 10^7^ ± 25.47^*b*^	6.58 × 10^7^ ± 16.09^*c*^	6.36 × 10^7^ ± 15.48^*c*^
Mid	8.59 × 10^7^ ± 30.27^*a*^	5.75 × 10^7^ ± 14.71^*c*^	7.28 × 10^7^ ± 21.85^*b*^	6.79 × 10^7^ ± 15.26^*b*^	7.41 × 10^7^ ± 20.77^*b*^	7.00 × 10^7^ ± 20.44^*b*^	5.30 × 10^7^ ± 14.44^*c*^	4.62 × 10^7^ ± 13.41^*c*^
High	7.75 × 10^7^ ± 14.08^*a*^	5.58 × 10^7^ ± 14.88^*b*^	7.16 × 10^7^ ± 15.64^*a*^	6.62 × 10^7^ ± 13.44^*a*^	7.28 × 10^7^ ± 19.92^*a*^	6.98 × 10^7^ ± 18.08^*a*^	2.27 × 10^7^ ± 13.96^*c*^	2.09 × 10^7^ ± 14.52^*c*^
**Bacterial abundance (cells/mL) in soil**								
	1.02 × 10^10^ ± 83.80^*a*^	2.61 × 10^9^ ± 21.83^*c*^	8.31 × 10^9^ ± 27.66^*b*^	2.8 × 10^9^ ± 19.99^*c*^	9.7 × 10^9^ ± 30.59^*b*^	4.0 × 10^9^ ± 24.14^*c*^	2.3 × 10^9^ ± 14.67^*c*^	1.1 × 10^9^ ± 9.19^*c*^

*Note:* S‐1 to S‐8; different mangroves stations. Low zone was within the mangroves ecosystem and mid zone was 200 m away from the mangroves (toward the open sea) while high zone was 400 m away from the mangrove ecosystem (toward the open sea). ± SD showing standard deviation while data (means ± SD, *n* = 9) followed by different letters (superscripts) indicate significant differences among variables (*a* = *p* < 0.05; *b* = *p* < 0.01; *c* = *p* < 0.001).

**FIGURE 5:**
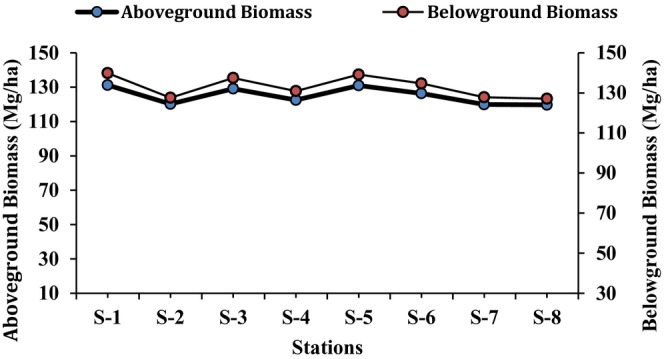
Comparison of above‐and belowground biomass of *A. marina* at eight stations (S‐1 to S‐8).

**FIGURE 6:**
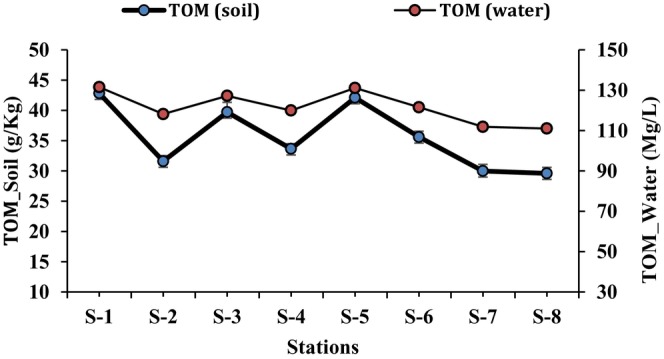
Comparison of total organic matter (TOM) in water and soil samples at eight stations (S‐1 to S‐8).

**FIGURE 7:**
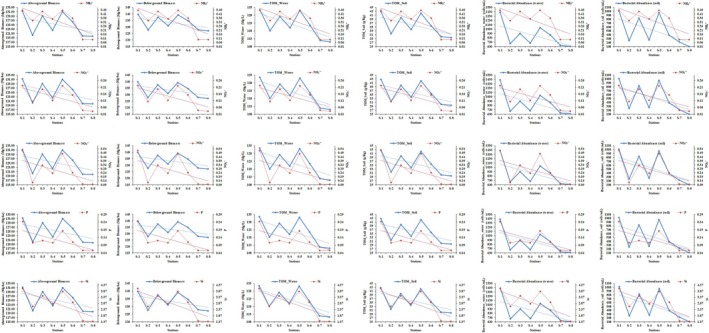
The comparison of the nutrients with biomass of mangroves, total organic matter (TOM) and bacterial abundance. On the basis of nutrients (${\mathrm{NH}}_{4}^{+}$, ${\mathrm{NO}}_{2}^{-}$, ${\mathrm{NO}}_{3}^{-}$, P and Si) availability; the biomass of the mangrove (above and below ground), total organic matter and bacterial abundance in the water samples of the mangrove ecosystem of 8 sites (1 to 8) are shown.

**FIGURE 8:**
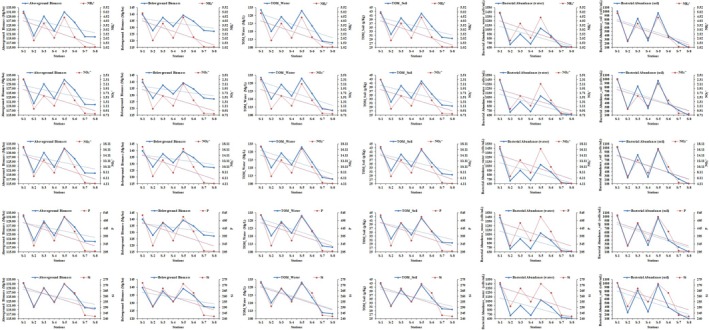
The comparison of the nutrients with biomass of mangroves, total organic matter (TOM) and bacterial abundance. On the basis of nutrients (${\mathrm{NH}}_{4}^{+}$, ${\mathrm{NO}}_{2}^{-}$, ${\mathrm{NO}}_{3}^{-}$, P and Si) availability; the biomass of the mangrove (above and below ground), total organic matter and bacterial abundance in the soil samples of the mangrove ecosystem of 8 sites (1 to 8) are shown.

*A. marina* was the only species found in all 8 sites. Therefore, this species was used to determine the biomass in this study. The biomass of the belowground and aboveground parts of *A. marina* was determined (Table 6). The results showed that the pattern of biomass of the belowground and aboveground parts of the plant ( *A. marina* ) depended on the concentration of nutrients found in these stations. The highest biomass (aboveground parts; 131.23 ± 2.09 Mg/ha, belowground parts; 139.86 ± 2.57 Mg/ha) of the plants was measured at station S‐1 (Figure 5). The proportion of aboveground and belowground parts of *A. marina* at station S‐1 was 48.41% and 51.59% respectively. The lowest biomass (aboveground parts; 119.72 ± 1.99 Mg/ha, belowground parts; 127.13 ± 2.01 Mg/ha) was recorded for the plants at station S‐8, while the percentage for the aboveground parts was 48.50% and for the belowground parts 51.50%. The order of biomass production of the plants was the same for all stations (S‐1 > S‐5 > S‐3 > S‐6 > S‐4 > S‐2 > S‐7 > S‐8), as was also the case for nutrients. The total organic matter in water and soil samples was also determined (Table 6). The results of organic matter showed that the highest concentration was recorded in both water (131.49 ± 2.25 mg/L) and soil (42.81 ± 1.56 g/Kg) from station S‐1, while the lowest organic matter values were recorded in the water (110.99 ± 2.02 mg/L) and soil (29.59 ± 0.95 g/Kg) samples from station S‐8 (Figure 6). The comparison of nutrients (both water and soil samples) with mangrove biomass, total organic matter (TOM) and bacterial abundance are also shown in the Figures 7 and 8.

**TABLE 6:** Comparison of above and belowground biomass of *A. marina* and total organic matter (TOM) in water and soil samples at eight stations (S‐1 to S‐8).

Stations	S‐1	S‐2	S‐3	S‐4	S‐5	S‐6	S‐7	S‐8
Aboveground (Mg/ha)	131.23 ± 4.79^*a*^	120.19 ± 2.43^*b*^	129.09 ± 2.32^*a*^	122.50 ± 1.95^*b*^	130.98 ± 1.74^*a*^	126.36 ± 1.93^*a*^	119.93 ± 1.97^*b*^	119.72 ± 1.92^*b*^
%	48.41	48.51	48.43	48.34	48.48	48.4	48.41	48.5
Below ground (Mg/ha)	139.86 ± 2.00^*a*^	127.59 ± 1.87^*b*^	137.47 ± 1.92^*a*^	130.93 ± 1.85^*a*^	139.17 ± 1.84^*a*^	134.71 ± 1.82^*a*^	127.83 ± 1.79^*b*^	127.13 ± 1.82^*b*^
%	51.59	51.49	51.57	51.66	51.52	51.6	51.59	51.5
TOM_soil (g/Kg)	42.81 ± 1.56^*a*^	31.59 ± 0.93^*b*^	39.71 ± 1.61^*a*^	33.64 ± 0.84^*b*^	42.09 ± 1.36^*a*^	35.59 ± 0.96^*a,b*^	29.98 ± 1.09^*b*^	29.59 ± 0.99^*b*^
TOM_water (Mg/L)	131.49 ± 1.31^*a*^	118.15 ± 1.29^*b*^	127.19 ± 1.46^*a*^	119.91 ± 1.40^*b*^	131.08 ± 1.24^*a*^	121.53 ± 1.42^*b*^	111.85 ± 1.32^*c*^	110.99 ± 1.46^*c*^

*Note:* ± SD showing standard deviation while data (means ± SD, *n* = 3_biomass, 9_TOM) followed by different letters (superscripts) indicate significant differences among variables (*a* = *p* < 0.05; *b* = *p* < 0.01).

## 4 Discussion

This is a comparative study between different mangrove sites in which the comparison between the nutrients and the biomass of the mangroves was determined. To assess the impact of nutrients on the bacterial community and the impact of bacteria on soil fertility, the abundance/quantification of bacteria was also determined. Nutrients promote the growth and productivity of mangroves, therefore the availability of nutrients leads to increase production of mangrove biomass (Alongi 2018). The nutrient concentration increases the microbial biomass (Kellogg and Levin 2022). Both nutrient availability and the microbial community are linked. These are responsible for increasing soil fertility, which in turn increases mangrove biomass production. The more nutrients are available, the higher the number of microbes. The microbial community accumulates in the soil where the decomposition of organic matter and the mobilization of nutrients take place, which helps to improve soil fertility and thus plant growth (Reef et al. 2010; Wang et al. 2017).

The results of soil samples from station S‐1 had the highest bacterial abundance as well as the highest nutrients (${\mathrm{NH}}_{4}^{+}$, ${\mathrm{NO}}_{2}^{-}$, ${\mathrm{NO}}_{3}^{-}$, P) concentration than the samples from station S‐8. This study also compared bacterial abundance in water and soil samples. Bacterial abundance was higher (significantly) in soil samples as compare to water samples, as soil provides a rich and diverse environment for microorganisms to thrive and has a greater capacity to support bacterial growth than water sources (Wu et al. 2021). Beside this, phosphorus (P) and Nitrogen (N) are considered the utmost important nutrients for increasing soil fertility as they are crucial for plant growth. Nitrogen plays a crucial role in the development of stems and leaves, while phosphorus is essential for root growth and seed production (Razaq et al. 2017). Present study showed that the soil of the mangrove ecosystem was enriched in phosphorus (P) and nitrogenous compounds (${\mathrm{NH}}_{4}^{+}$, ${\mathrm{NO}}_{2}^{-}$ and ${\mathrm{NO}}_{3}^{-}$). Phosphorus is crucial for plant biomass production as it's a key component of energy transfer molecules (like ATP and ADP), nucleic acids (DNA and RNA), and various metabolic processes, ultimately impacting photosynthesis, cell division, and overall plant growth (Khan et al. 2023). Among the three forms of nitrogen, the nitrate (${\mathrm{NO}}_{3}^{-}$) is the utmost suitable nitrogen form for the plant growth; as most plants readily absorb it directly from the soil without the need for further transformation/conversion. It regulates various aspects of plant metabolism and development (Camut et al. 2021). Therefore, for the plants growth, ${\mathrm{NO}}_{3}^{-}$ is the preferred choice, compared to ${\mathrm{NH}}_{4}^{+}$ or ${\mathrm{NO}}_{2}^{-}$ (Zayed et al. 2023). The comparatively higher amount of ${\mathrm{NO}}_{3}^{-}$ in the present soil samples indicated that the soil of the mangrove sites (S‐1 to S‐8) was fertile. The highest concentration of P and ${\mathrm{NO}}_{3}^{-}$ was investigated at site S‐1, therefore the highest biomass was calculated at this site. Silicates play a crucial role in plant biomass production by enhancing growth, improving stress tolerance, and promoting nutrient uptake (Khan 2024). It also plays a significant role in enhancing phosphorus absorption in the soil, making it more available for the plants (Greger et al. 2018). Previous literature (Sahebi et al. 2015; Raza et al. 2023; Khan 2024) indicated that significant amount of ${\mathrm{SiO}}_{3}^{2-}$ (= Si) could be sufficient for phosphorus uptake by the plants. As compared to other sites, site S‐1, had highest concentration of silicate therefore, the biomass of mangroves was significantly higher at this site.

Previous literature (Leff et al. 2015; Dai et al. 2022; Yuan et al. 2022; Cui et al. 2021) indicated that the microbial population can thrive and multiply under nutrient enriched medium (environment). The present findings revealed that bacterial count was also a nutrient‐dependent phenomenon. The highest nutrient levels were recorded at station S‐1, while the lowest nutrient levels were recorded at station S‐8. Therefore, the maximum bacterial count was found in station S‐1 and the lowest in station S‐8. The order of the total bacterial count for all stations was: S‐1 > S‐5 > S‐3 > S‐6 > S‐4 > S‐2 > S‐7 > S‐8. In addition, the bacterial communities have a remarkable contribution in recycling of nutrients and for promoting the plants growth (Chen et al. 2016). The bacterial community also solubilize silicon, which becomes more readily available to the plants. Si increases phosphorus absorption in soil (As previously mentioned), reducing the soils' ability to bind phosphorus, making it more readily accessible for the plants uptake (Greger et al. 2018). Furthermore, phosphorus‐solubilizing bacterial communities also significantly improve soil fertility by converting insoluble forms of phosphorus into a readily available form that plants can easily take up. Therefore, these bacterial communities enhance nutrients uptake and plant growth by facilitating the release of essential nutrients trapped in the soil matrix; they essentially act as “nutrient mobilizers” by altering the soils' chemical environment (Alori et al. 2017; Verma et al. 2022; Etesami and Schaller 2023; Thepbandit and Athinuwat 2024). It has also been investigated that the microbial population diversity plays a vital role in the cation bridges, ionization of silicic acid, and carboxylation, which highly support the assimilation of Si in the rhizosphere (Khan 2024). Thus, a combination of Si/${\mathrm{SiO}}_{3}^{2-}$ and microbial activity could play a remarkable role in enhancing soil fertility, which in turn promotes plant growth. The findings of the present research showed that the highest concentration of Si and microbes in station S‐1 supported the production of mangrove biomass.

To determine the effect of nutrients on mangroves growth, the total biomass production of the mangrove plants at all stations was determined. As *A. marina* was the only species present at all sites, the biomass of this species was determined. The order of biomass was: S‐1 > S‐5 > S‐3 > S‐6 > S‐4 > S‐2 > S‐7 > S‐8, the order of nutrients concentration (for all the sites) was also same. The results supported our hypothesis that “the amount of biomass produced by a plant was directly linked to the availability of the nutrients”. A soil with a higher nutrient content enables greater biomass production, while low nutrient availability significantly reduces biomass (Yan et al. 2016; Schröder et al. 2018; Furey and Tilman 2021). The comparison of above‐ and belowground biomass of *A. marina* from all stations was also determined. The present findings revealed that the biomass (both above and below ground) of *A. marina* from site S‐1 was the highest overall, while the biomass of S‐8 site was the lowest. The order of biomass production of the plants was the same as for nutrient availability. The results also showed that below‐ground biomass was higher than above‐ground biomass. It was suggested that the majority of a mangrove tree's biomass is stored in its extensive root system rather than in its aboveground parts (stem, branches and leaves); this is due to adaptation to the tidal and saline environments in which they inhabit (Soares and Schaeffer‐Novelli 2005; Srikanth et al. 2016; Meng et al. 2021).

A microbial population is highly considered a form of biomass production, as the collective mass of microorganisms within a community constitutes the microbial biomass (Young and Unc 2023). In the present study, bacterial biomass was investigated in seawater and soil samples. It was found that bacterial biomass was higher in the soil samples of mangroves than in the water samples. This was as a result of the high organic matter ratio in the soil, which resulted in more diverse microbial community (Gómez‐Acata et al. 2023). In addition, the bacterial biomass in water samples from the mangrove ecosystem was compared with the bacterial biomass outside the mangrove ecosystem (toward the open sea). The results showed that the bacterial biomass in the open seawater is relatively low. Because the nutrient concentration was highest in the mangrove ecosystem, which led to a rise in bacterial biomass. The comparison of the total organic matter of the soil and water samples of the mangrove ecosystem from all sites was also investigated. The findings revealed that the total organic matter in the soil samples of the mangrove ecosystem was significantly higher than the organic matter in the water samples. The mangrove soils have a higher capability to accumulate and trap organic matter in their sediment layers, making the soil much richer in organic matter compared to the water (Tang et al. 2023).

The findings of this study help to understand the impact of nutrient dynamics on mangrove health and resilience. The mangrove ecosystem is a sustainable ecosystem as it is a hub for biodiversity and a major source of fish and shellfish farming, and thus can support the blue economy. In addition, mangroves restore large amount of environmental carbon in the form of biomass, which means that the mangrove ecosystem reduces pollution. In this study, the sustainability of the mangrove ecosystem has been described, which highlighted the importance of mangrove forests. In order to maintain a sustainable environment, the government and non‐governmental organizations should take a step to restore the mangrove ecosystem.

### 4.1 Significance and Limitation of the Study

The comparison of nutrients and the biomass of mangrove plants and bacterial abundance was investigated in this study. It was determined that bacterial abundance and biomass of mangrove plants depended on the availability of the nutrients. This study provides important insights into biomass production through nutrient availability. The analysis of nutrients also revealed that there was a problem of eutrophication in the mangrove sites. The excessive amount of nutrients in a water body is called eutrophication. It can cause considerable harm to the mangrove plants and damage the pneumatophores by causing algal blooms that block sunlight from reaching the mangrove roots, leading to reduced oxygen levels in the water (hypoxia), impacting the mangrove's ability to thrive and potentially causing die‐offs due to stress and lack of oxygen. This can disrupt the entire mangrove food web and alter the species composition within the ecosystem (Mandura 1997; Feller et al. 1999, 2017; Lovelock et al. 2009; Huxham et al. 2010; Hayes et al. 2020). In the present study, the effects of eutrophication on the mangrove plants were not investigated. This is the potential limitation of this study. In the next part of our study, we will investigate the effects (long term) of eutrophication on mangrove forests.

## 5 Conclusion

This study investigated the role of mangroves in nutrient cycling. The mangrove ecosystem is the main platform for the absorption of nutrients and basic elements (released by anthropogenic and natural activities) in the soil. The nutrients directly lead to an increase in biomass production of the microbial community. This microbial community, increases soil fertility and thus increases plant growth, leading to higher biomass production of the mangroves. Therefore, the relationship between nutrients and the microbial community may be a better measure for assessing mangrove biomass production. The mangrove ecosystem also serves as a nursery area for fish and shellfish and is therefore a major food source for most marine organisms and also for human consumption. Moreover, the mangrove forests convert a large amount of inorganic carbon into the useful organic form therefore the mangroves create a healthy environment. In this way, this study is in line with SDGs 12 (responsible consumption and production of food), 13 (climate action), 14 (life below water), and 17 (partnerships with the goals). It is concluded that “mangrove forests play an important role in blue carbon sequestration”. Therefore, policy makers can use these research results as future strategies for the sustainable management of mangrove forests.

## Author Contributions

**Sadar Aslam:** data curation (lead), formal analysis (lead), investigation (lead), methodology (lead), resources (equal), software (lead), writing – original draft (lead), writing – review and editing (equal). **You‐Shao Wang:** conceptualization (equal), funding acquisition (lead), investigation (lead), project administration (lead), resources (equal), supervision (lead), validation (equal), visualization (lead), writing – review and editing (equal).

## Ethics Statement

In this study, no live animal has been used. All necessary permits for sampling and observational field studies have been obtained by the authors from the competent authorities. The study is compliant with CBD and Nagoya protocols.

## Conflicts of Interest

The authors declare no conflicts of interest.

## Supporting information


**Figure S1.** Calibration curve of nutrients ammonium (A), nitrite (B), nitrate (C), phosphate (D), and silicate (E).
**Figure S2.** Flow cytometric images for the abundance of bacteria in seawater sample in low, mid, and high zones of eight stations (S‐1 to S‐8).
**Figure S3.** Flow cytometric images for the abundance of bacteria in soil samples of eight stations (S‐1 to S‐8).

Appendix S2.

## Data Availability

The data that supports the findings of this study are available in the Supporting Information of this article.
