# Supplementary material for: Determination of Nutrients, Biomass, and Bacterial Quantification in Different Mangroves Sites: A Comparative Study on Nutrients Dependent Biomass Production
Source: Ecol Evol. 2025 Jul 4;15(7):e71697. doi: 10.1002/ece3.71697 (PMC12231210; doi:10.1002/ece3.71697)

**Supplementary Materials**

**Preparation of reagents and standard solutions for analysis**

Analytical grade chemicals/reagents were used which were purchased from Shanghai Aladdin Biochemical Technology Limited Company. Stock standard solutions for nitrite (NO_2_^−^), nitrate (NO_3_^−^) and ammonium (NH_4_^+^) were prepared by dissolving KNO_3_, KNO_2_ and NH_4_Cl, respectively in deionised distilled water and stored in the freezer at −20 ℃. Working solutions were prepared, in KCl (2 M) from the stock standard solutions, in the range of 0.1-1.0 g/mL for NO_2_^-^, 1.0-5.0 g/mL for NO_3_^-^ and 0.1-1.2 g/mL for NH_4_^+^. Chromogenic reagent (Chr-R) was prepared by mixing 1.5 g sulphanilamide, 0.225 g N-(l-naphthyl) ethylenediamine dihydrochloride (NED), 0.5 g poly (vinyl alcohol) (PVA) and 25mL HCl (37%) in 100 mL of deionised distilled water. Sodium citrate (2.5%) was a carrier solution.

For the analysis of NH_4_^+^, the samples were oxidized. For the oxidation, the samples were treated with 0.08 M solution of NaClO and 1.5 M solution of KBr and 1 M of NaOH (Pasquali et al., 2007). Copper sulphate (CuSO_4_), sodium citrate (Na_3_C_6_H_5_O_7_) and potassium chloride (KCl) of analytical quality were used for interference studies. For the validation of NH_4_^+^, a solution containing 3.9065 g of sodium salicylate (C_7_H_5_NaO_3_) and 0.0625 g of sodium nitroprusside (C_5_FeN_6_Na_2_O) with 25 mL distilled water was prepared, using Berthelot reaction. Buffer solution was prepared by mixing 2.96 g of sodium hydroxide (NaOH), 9.96 g of disodium hydrogen phosphate (Na_2_HPO_4_) adding 10 mL sodium hypochlorite (NaClO), and diluting to 100 mL with distilled water. Nitrate (NO_3_^-^) was reduced to nitrite (NO_2_^-^), using a copperized cadmium column to be analysed as nitrite (NO_2_^-^).

For the analysis of phosphate (PO_4_^3-^), the standard solution was prepared by adding 1 mL of ammonium molybdate ((NH_4_)_6_Mo_7_O_24_) and 0.4 mL of hydrazine sulphate ([N_2_H_5_]^+^[HSO_4_]^−^) and 10 mL of deionised distilled water added. The measuring flasks containing this mixture, were kept for 30 min in a water bath for heating at 60 ℃. A blue colour was developed, indicating ammonium phosphomolybdate complex was formed. When the solution was cooled down then its absorbance was measured at the wavelength of 830 nm. A blank solution was prepared and used for creating the accuracy in the results.

**Seawater and soil samples collection and preparation for analysis**

To determine the importance of the mangrove ecosystem, we divided each station into three zones (low, mid and high) and compared the bacterial abundance at each station in the three zones. The low zone was within the mangrove ecosystem, the mid zone was 200 meters away from the mangroves (towards the open sea), while the high zone was 400 meters away from the mangrove ecosystem. For the collection of seawater samples, sterilized amber glass bottles (500 mL) were used. After collection, the samples were acidified with 50 μL of 10.2 M HCl and stored in an icebox during transportation. In our laboratory, these samples were stored in the dark at 4 ℃ for no longer than 3 days. The soil samples were collected (0-20 cm) and placed in the zip-locked polythene bags. Sampling transportation was done using an ice box. The samples were stored at 4 ℃ in the laboratory and the analysis was performed earlier to avoid possible loss of analytes.

For the analysis, the soil samples were dried for 24 h at room temperature and sieved (2 mm grain size). Approximately 5 g of the dried soil was placed in a plastic bottle and mixed with 50 mL of 2M KCl. A suspension was formed which was mechanically shaken and after shaking for 90 min it was finally centrifuged (200 rpm) and filtered through a 0.45 um polysulfone membrane. The supernatant was used for analysis (Pasquali et al., 2007).

About 250 μL of the sample was reacted with NaOH, NaClO and KBr solutions, and ammonium (present in the sample) was oxidized to nitrite. Then this nitrite containing solution was treated with the chromogenic reagent (Chr-R). The absorbance was measured at a wavelength of 543 nm.

**Determination of nitrite (**NO_2_^−^**), nitrate (**NO_3_^−^**) and ammonium (**NH_4_^+^**)**

For the analysis of NO_2_^−^, NO_3_^−^ and NH_3_, the sample was injected in the system where sodium citrate (Na_3_C_6_H_5_O_7_) solution was used as a carrier. 1) For the analysis of nitrite (NO_2_^−^), the sample was treated with the chromogenic reagent (Chr-R) and blue colour was developed. The absorbance was measured by the spectrophotometer. 2) For the analysis of nitrate (NO_3_^−^), the sample was reacted with a mixture solution of NH_4_Cl and EDTA then this sample was passed through the reduction column (Cd comprised). In the reduction column, the nitrate (NO_3_^−^) was reduced to nitrite (NO_2_^−^), and then absorbance was measured. 3) For the analysis of ammonium, the sample was treated with of NaOH, NaClO and KBr solutions and in the oxidation column NH_3_ oxidized to NO_2_^−^. After oxidation, the sample mixture was treated with the Chr-R and the absorbance was measured. A Hoffman key was placed in this channel to reduce and control the flow of nitrite.

**Determination of phosphate in seawater and soil samples**

Seawater sample was filtered through Whatman filter paper (0.45µm) and used for phosphate analysis. For the analysis of soil nutrients; the samples were digested first and then filtered and further used for PO_4_^3-^ analysis. For the digestion process of the soil samples, 2 g of soil sample was taken in a 250 mL volumetric flask and heated (130 ℃) in a hot plate by adding 20 mL of conc. HNO_3_ and mixed well. The organic matter in the sample was heated to almost 130 ℃ to reach oxidation. The sample was heated until the dark colour (because of organic matter in the soil) of the soil sample was completely disappeared. After heating, the sample mixture (soil-HNO_3_) was cooled slightly. In fuming hood, this sample mixture was treated with 30 mL perchloric acid (HClO_4_) and then the sample was heated at 200 ℃ for 20 min. A little extra perchloric acid was added to wash down the black particles which were fixed to the edges of the flask. Then, the mixture was cooled to at room temperature for 10–15 min, and was relocated to a volumetric flask (250 mL) then diluted with deionised distilled water made up to the mark. This sample mixture was left (to settle down the particles) and aliquot was taken for analysis. About 1 mL of ammonium molybdate ((NH_4_)_2_MoO_4_) and 0.4 mL of hydrazine sulphate (H_4_N_2_.H_2_O_4_S) were added in the aliquot. Finally, this sample mixture was diluted with deionized distilled water (10 mL) and heated a water bath for 30 min at 60 °C. After 30 min, the blue colour was appeared, then the solution was cooled at room temperature and the absorbance was measured (830 nm).

**Determination of silicon in the water and soil samples**

All reagents/chemicals were of analytical grade. The water used for the preparation of the reagent was filtered/purified using a Milli-Q plus Millipore system (Millipore Bedford, USA). Water samples were analysed without sample preparation steps. For the preparation of soil samples, 10 g of soil was weighed, dried at 65 ℃ until the constant weight was obtained and then finely ground. To avoid any contamination, polypropylene tubes and bottles were used to prepare the samples and standard solutions. Now these ground soil samples were further processed involving digestion/extraction. The procedure for the extraction of Si from the soil samples was started by the addition of 100 mL of acetic acid (0.5 mol/L). During extraction the tubes were shaken horizontally for 1 h (50 rpm) at room temperature. So, as to settle down the soil particles, the tubes were left for 15 min. After that, the solution was filtered and kept inside the flasks for 12 h period before analysis (Carneiro et al., 2007).

About 5 mL of the test and reference solutions were taken in the 100 mL volumetric flasks then 5 mL of 5% ammonium molybdate solution was added in each of the flask and mixed well, finally left for 8-10 min. The yellow colour was appeared. 5 mL of 10% tartaric acid was added, and mixed. These mixture solutions were left for 1-2 min and 20 mL of recovery solution were taken and deionised distilled water was added to the flask up to the mark (100 mL), which was then mixed by using vortex. The volume of the solution is adjusted to the mark with water, stirred, left for 2 h. Standard solutions were prepared according to standard protocol and were kept for 2 h at room temperature. The absorbance of the solutions was measured by the spectrophotometer at the wavelength of 830 nm.

**Determination of Bacterial count by Flow cytometric (FCM) technique**

**Sample Collection and Preparation**

The soil samples were collected from the mangroves area (0-20 cm). Immediately after collection, the samples were packed in zip-locked polyethylene bags. In the laboratory (within 24 h of collection), the samples were prepared for flow cytometric analysis. For this purpose, 3 g of the wet samples were transferred into 20 mL sterilized glass vials containing 10 mL of a solution of 2 % paraformaldehyde and 0.1 % sodium pyrophosphate (Na_4_P_2_O_7_). All vials were stored in the dark at 4 ℃ until analysis.

The soil samples bacteria, in order to detached the bacterial cells from the samples, 2 g of wet soil sample was taken and 5 mL of saline solution (1%) was added and the soil was treated with the ultrasonic probe (3 × 20 s with a 20 s break in between), by its flat tip. This treatment was given for 1 min while the power of 38 W was supplied. After ultrasonic treatment, the bacterial cells were detached from the soil, the subsample was then homogenized by vortexing. 1 mL of the subsample was taken in the sterile reaction tube and 0.5 mL Histodenz solution (1.3 g/mL, Sigma-Aldrich) was added in the sample. This tube was centrifuged (90 min at 4 ℃ and 17135 × g). After centrifugation, two layers were appeared in the tube; the lower layer (containing Histodenz) was discarded while the upper layer was used for further analysis (Frossard et al., 2016).

The water samples were collected from the surface layer, using sterile plastic bottles, then immediately preserved with paraformaldehyde (0.5% ﬁnal concentration) and frozen in the liquid nitrogen. The samples were kept at -80 ℃ until analysis.

**S1.** Calibration curve of nutrients ammonium (A), nitrite (B), nitrate (C), phosphate (D) and silicate (E)


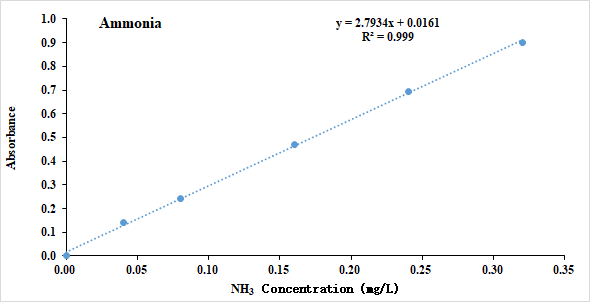


**A**


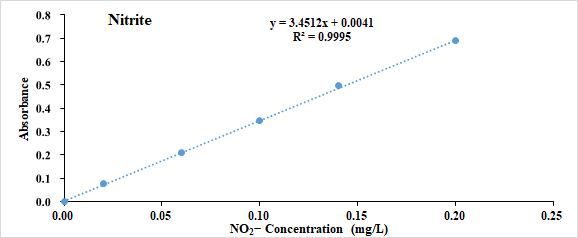


**B**


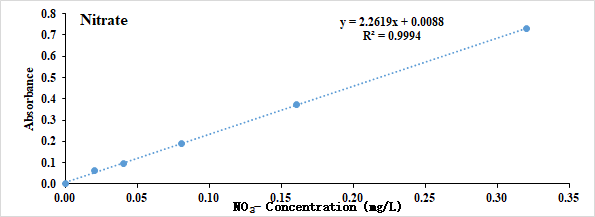


**C**


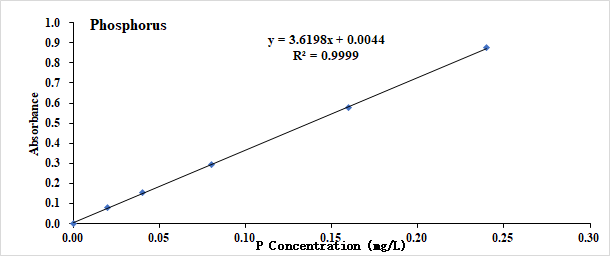


**D**


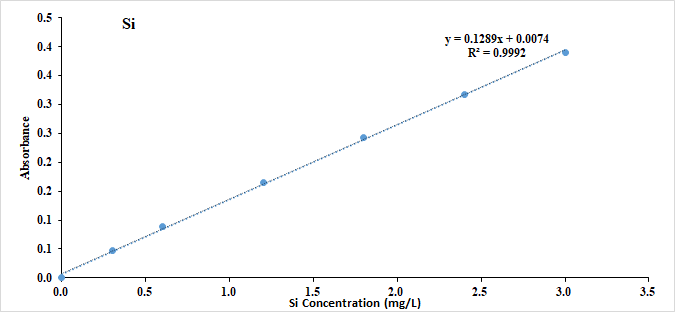


**E**

**S2 (1-8).** Flow cytometric images for the abundance of bacteria in seawater sample in low, mid and high zones of eight stations (S-1 to S-8)

**S2-1**. Abundance of bacteria in low (A), mid (B) and high (C) zones of station S-1


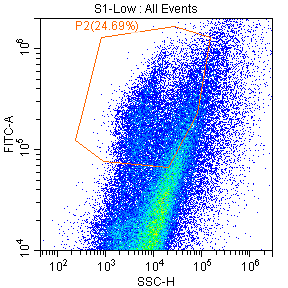

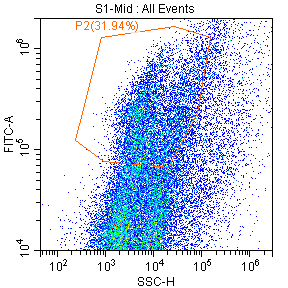

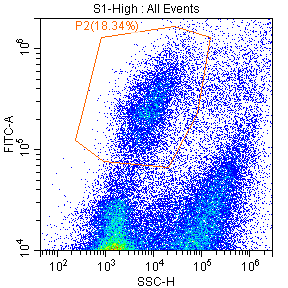


**A**

**C**

**B**

**S2-2. Abundance of bacteria in low (A), mid (B) and high (C) zones of station S-2**


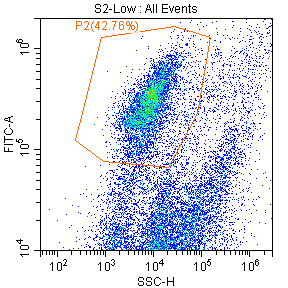

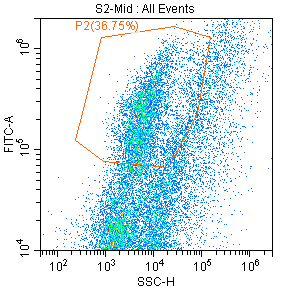

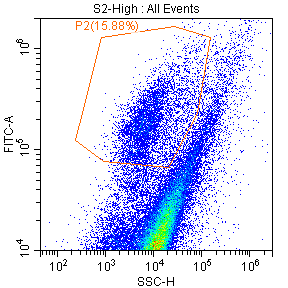


**A**

**C**

**B**

**S2-3. Abundance of bacteria in low (A), mid (B) and high (C) zones of station S-3**


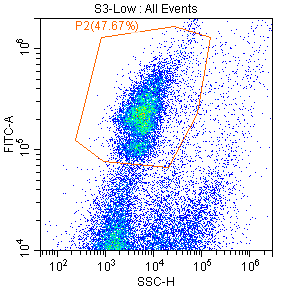

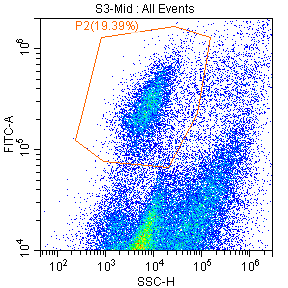

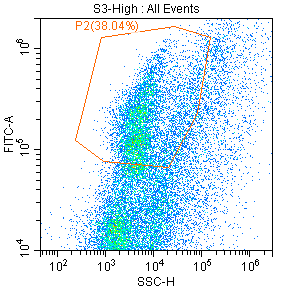


**A**

**B**

**C**

**S2-4. Abundance of bacteria in low (A), mid (B) and high (C) zones of station S-4**


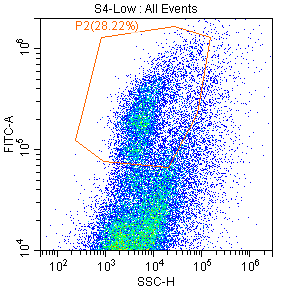

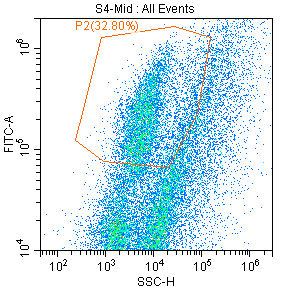

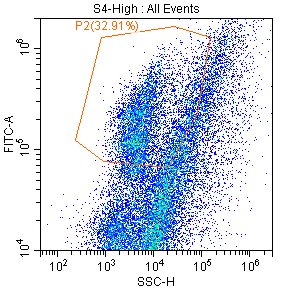


**A**

**C**

**B**

**S2-5. Abundance of bacteria in low (A), mid (B) and high (C) zones of station S-5**


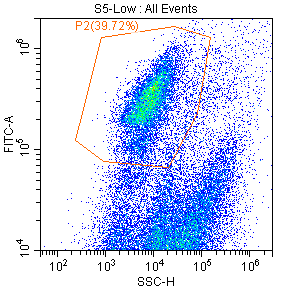

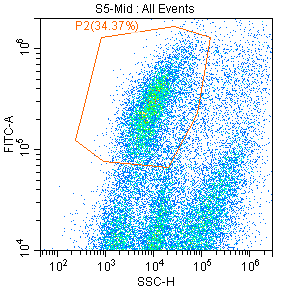

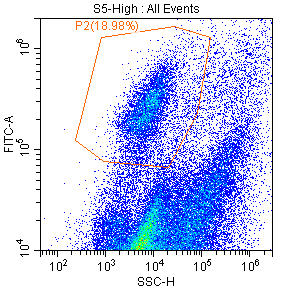


**A**

**C**

**B**

**S2-6. Abundance of bacteria in low (A), mid (B) and high (C) zones of station S-6**


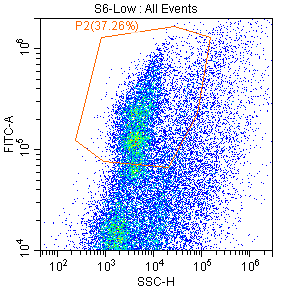

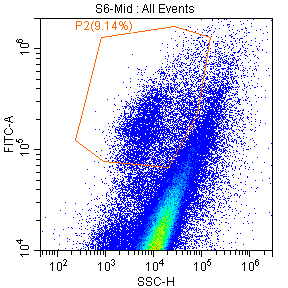

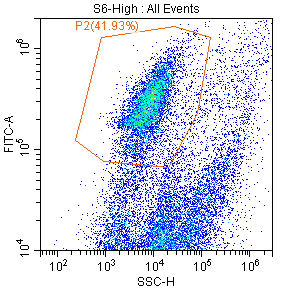


**A**

**C**

**B**

**S2-7. Abundance of bacteria in low (A), mid (B) and high (C) zones of station S-7**


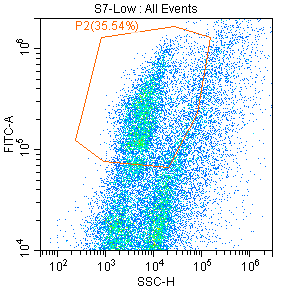

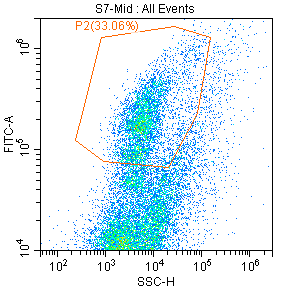

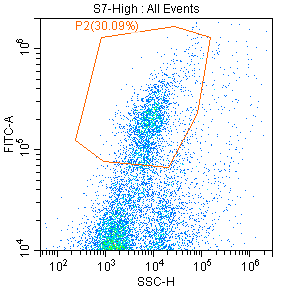


**A**

**C**

**B**

**S2-8. Abundance of bacteria in low (A), mid (B) and high (C) zones of station S-8**


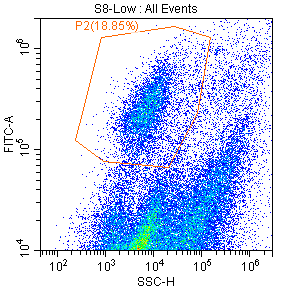

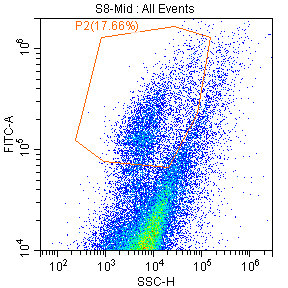

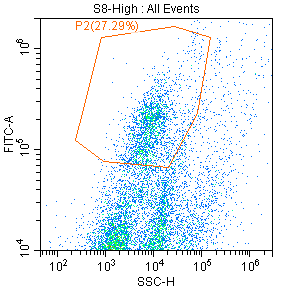


**A**

**C**

**B**

**S3. Flow cytometric images for the abundance of bacteria in seawater samples**

**S3-1. Abundance of bacteria in soil samples of station S-1**


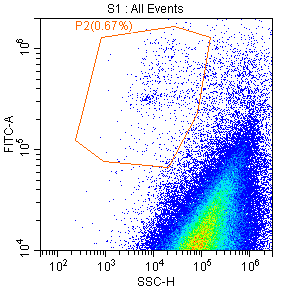


**S3-2. Abundance of bacteria in soil samples of station S-2**


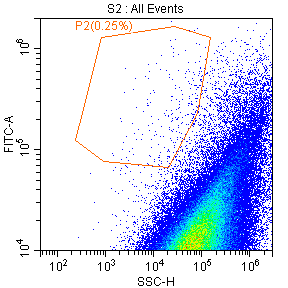


**S3-3. Abundance of bacteria in soil samples of station S-3**


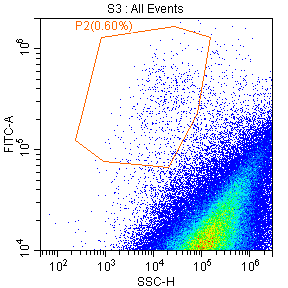


**S3-4. Abundance of bacteria in soil samples of station S-4**


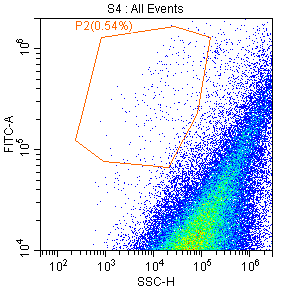


**S3-5. Abundance of bacteria in soil samples of station S-5**


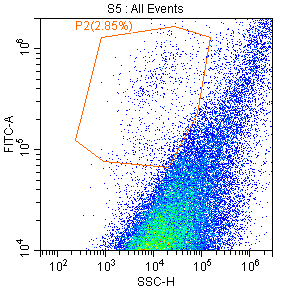


**S3-6. Abundance of bacteria in soil samples of station S-6**


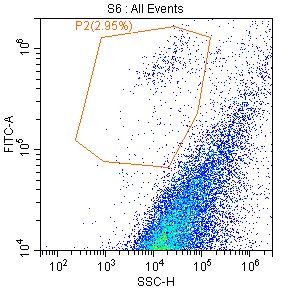


**S3-7. Abundance of bacteria in soil samples of station S-7**


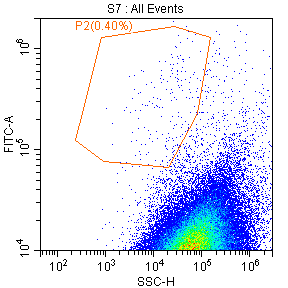


**S3-8. Abundance of bacteria in soil samples of station S-8**


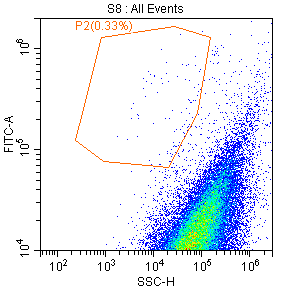

Supplement: Supplementary file 1 — Figure S1. Calibration curve of nutrients ammonium (A), nitrite (B), nitrate (C), phosphate (D), and silicate (E). Figure S2. Flow cytometric images for the abundance of bacteria in seawater sample in low, mid, and high zones of eight stations (S‐1 to S‐8). Figure S3. Flow cytometric images for the abundance of bacteria in soil samples of eight stations (S‐1 to S‐8). [file ECE3-15-e71697-s002.docx]
